# Pharmacological Benefits and Risk of Using Hormones in Organ Perfusion and Preservation Solutions in the Aspect of Minimizing Hepatic Ischemia-Reperfusion Injury during Storage

**DOI:** 10.1155/2019/6467134

**Published:** 2019-11-11

**Authors:** Aneta Ostróżka-Cieślik, Barbara Dolińska

**Affiliations:** ^1^Department of Pharmaceutical Technology, Faculty of Pharmaceutical Sciences in Sosnowiec, Medical University of Silesia in Katowice, Sosnowiec 41-200, Poland; ^2^“Biochefa” Pharmaceutical Research and Production Plant, Sosnowiec 41-200, Poland

## Abstract

For several years, research has been carried out on the effectiveness of solutions for perfusion and preservation of organs, including the liver. There is a search for an optimal pharmacological composition of these solutions, allowing to preserve or improve vital functions of the organ for as long as possible until it is transplanted into a recipient. Hormones due to their properties, often resulting from their pleiotropic effects, may be a valuable component for optimizing the composition of liver perfusion and preservation solutions. The paper presents the current state of knowledge on liver perfusion and preservation solutions modified with hormones. It also shows the characteristics of the hormones evaluated, taking into account their physiological functions in the body.

## 1. Introduction

Transplantation is currently the only method of treating patients with end-stage liver failure. However, the organ shortage and limited time of graft survival outside the donor organism are a challenge for contemporary transplantology. One of the directions of obtaining grafts is the extension of criteria to accept marginal organs, including those from older people. Effective protection of hepatocytes against ischemia-reperfusion injury is therefore a challenge in the aspect of undertaking proper vital functions by the organ. For several years, research has been carried out on the effectiveness of solutions for perfusion and preservation of organs, including the liver. There is a search for an optimal pharmacological composition of these solutions, allowing to preserve or improve vital functions of the organ for as long as possible until it is transplanted into a recipient. Hormones due to their properties, often resulting from their pleiotropic effects, may be a valuable component for optimizing the composition of liver perfusion and preservation solutions. They can minimize the risk of graft dysfunction and influence the degree of hepatocyte damage during cold ischemia. The paper presents the current state of knowledge on liver perfusion and preservation solutions modified with hormones. It also shows the characteristics of the hormones evaluated, taking into account their physiological functions in the body.

## 2. Literature Search

A systematic review of the literature was carried out in accordance with the Preferred Reporting Items for Systematic Reviews and Meta-Analyses (PRISMA). The databases such as Medline, PubMed, Cochrane, and Embase had been searched until May 31, 2019. They were searched for articles on the effectiveness of liver perfusion and preservation solutions modified with hormones. Review papers and the reference lists of papers were also browsed to identify additional articles. The review also includes studies published between January 1, 1998, and May 31, 2019. Each article was evaluated by two authors using a structured assessment tool. Articles in English relating to studies using animal liver perfusion and preservation models, in which each species, age, sex, race, and sample size were taken into account, were included. There were no prospective, multicentre, or randomized studies with a control group carried out in humans. Studies in which the hormone was administered to the animal and/or directly to the graft at any stage of the experiment in the form of injection and/or infusion or in the diet were excluded. Only preservation solutions registered and commonly used in the liver transplantation were accepted. The terms “organ preservation solutions, therapeutic use, hormones, additives, pharmacological agent, trophic factors, perfusion, ischemia-reperfusion, liver transplantation, steatotic liver models” from Medical Subject Headings (MeSH) and Emtree (Elsevier's Life Science Thesaurus) were applied with logical operators (AND, OR, and NOT).

Initially, the literature review included 777 articles related to the topic of the analysed studies. After applying the inclusion/exclusion criteria, 25 articles were qualified for the evaluation (Table 1).

## 3. Preservation Solutions

Ischemia-reperfusion injury (IRI) results in mitochondrial damage, disturbed energy metabolism, increased reactivity of free oxygen radicals (ROS), and the release of inflammatory cytokines in the liver [26–28]. To limit the occurrence of these processes, livers are stored in preservation solutions. UW (University of Wisconsin) is the most commonly used solution. It has been recognized as the gold standard for transplantology. It allows the liver to be stored for up to 12 hours by simple hypothermia. It is an intracellular fluid due to the high content of potassium (125 mmol/l) and the low content of sodium (25 mmol/l). Its disadvantages include high viscosity, which reduces perfusion efficiency, and high concentration of K^+^ ions, which requires a preflush of liver grafts before reflow in the recipient [29]. The components that guarantee its effectiveness are lactobionate, raffinose, and glutathione. The other components have a marginal effect; therefore, it is assumed that they may be omitted. Insulin, whose action is controversial, was also introduced into the fluid composition [30]. IGL-1 (Institute George Lopez) solution was developed based on UW. The content of sodium and potassium ions was changed, and an extracellular fluid with a high Na^+^ (120 mmol/l) and low K^+^ (25 mmol/l) concentration was developed. The risk of cardiovascular complications was minimized in this way [31]. It has been found that in comparison with UW, it shows a more beneficial effect in the protection of fatty livers. It protects against oxidative stress, mitochondrial damage, and alterations in vascular resistance [32]. HTK (Bretschneider's solution, Custodiol) is an intracellular fluid, crystalloid, used to store the heart, liver, kidneys, and pancreas [33]. Its task is to counteract the retention of sodium and calcium ions in the intracellular space and buffer the extracellular space by means of the histidine/histidine HCl system during organ ischemia [34]. In turn, Ringer's solution (in combination with heparin) is used to remove residual blood in the collected organ. In the next stage of transplantation procedures, the graft is stored in a suitable preservation solution. Fluid compositions that were modified with hormones are shown in Table 2.

## 4. Hormonal Additives

### 4.1. Melatonin

Melatonin (*N*-acetyl-5-methoxytryptamine) is a hormone synthesized mainly in pinealocytes, and to a lesser extent in the retina, Harder's lacrimal glands, gastrointestinal tract, bone marrow cells, platelets, and lymphocytes. Its secretion depends on the diurnal cycle: light/darkness. The maximum concentration of melatonin in the blood (20–100 pg/ml) is observed during the night hours, between 2:00 and 4:00. The half-life of endogenous and exogenous melatonin in the blood is 30–60 minutes and 12–48 minutes, respectively [35–37]. The highest concentration of melatonin was determined in the hepatobiliary system (2000–11000 pg/ml) [38]. It probably protects intestinal epithelial cells from damage caused by bile acids [39]. This hormone is also found in the cerebellum, respiratory epithelium, kidneys, endothelial cells, thyroid, and ovaries [40]. The molecule of this hormone is characterized by lipophilic nature and low molecular mass (232 Da), owing to which it easily penetrates the cell membranes and diffuses into the physiological fluids of the body. Melatonin acts on target cells both by means of receptors and independently of them [41]. It has the ability to bind to intracellular proteins, receptors located in the cell membrane, and nuclear receptors. The melatonin MT1 (MTNR1A) and MT2 (MTNR1B) membrane receptors are conjugated to G proteins and are made up of 7 transmembrane hydrophobic regions with a helical structure. Nuclear receptors, in turn, belong to the ROR/RZR (retinoid orphan receptors/retinoid Z receptors) subfamily. RZR-*β* is present in the nervous system cells and RZR-*α* in B lymphocytes, rat spleen, and thymus cells, as well as in hepatocytes, smooth muscle cells, and testicular cells of laboratory rodents [42–45]. It has been found that high-affinity melatonin binding sites are located in the hepatocyte nuclei [46]. Melatonin has antioxidant, angiogenetic, anti-inflammatory, immunostimulatory, and anticancer properties. It has the ability to neutralize free oxygen radicals and stimulate the action of antioxidant enzymes, i.e., superoxide dismutase, catalase, and glutathione peroxidase [47–50]. It inhibits lipid peroxidation *in vivo* more effectively than vitamin C and vitamin E [51]. It shows a synergistic effect with antioxidants, preventing the formation of free oxygen radicals [52].

Zaouali et al. conducted studies on the efficacy of melatonin in the protection of hepatocytes in an *ex vivo* model of isolated rat steatotic livers. Melatonin was added to the IGL-1 solution at a dose of 100 *μ*mol/L, and its activity was evaluated in relation to Ringer's lactate, IGL-1, and UW solutions. The livers were stored in the solutions for 24 h (4°C) and then reperfused for 2 h (37°C). The extent of liver damage was assessed in each group and melatonin proved to have positive effects. The livers rinsed with IGL-1 with the addition of melatonin showed the lowest levels of released alanine aminotransferase (ALT) and aspartate aminotransferase (AST), high bile production, and high BSP clearance (sulfobromophthalein (BSP) clearance). There was also increased induction of NO by activation of constitutive nitric oxide synthase (eNOS) and reduced vascular resistance. Melatonin reduced the mitochondrial oxidative stress and increased the respiratory chain activity [1]. In another publication, Zaouali et al. analysed the effect of melatonin (100 *μ*mol/L) and trimetazidine (10–3 *μ*M/L) addition to the IGL-1 solution in the modulation of ER (endoplasmic reticulum) stress and autophagy in steatotic liver grafts through activation of AMPK. They assessed ER stress (GRP78, PERK, and CHOP) and autophagy (beclin-1, ATG7, LC3B, and P62). They found a significant decrease in GRP78, pPERK, and CHOP activation after reperfusion. The inhibition of AMPK induced an increase in ER stress and a significant reduction in autophagy [2]. Gunal et al. modified the UW solution with melatonin at 130 *μ*mol/L. They examined the solution effectiveness in the Wistar rat liver transplantation model. They found that melatonin had a protective effect on Kupffer cells. The amount of enzymes released (LDH, AST, and ACP) was significantly lower compared to the control group. There was also an increase in the expression of heat-shock proteins HSP 70, which play an important role in maintaining normal homeostasis and reducing lipid peroxidation [3]. The research suggests that melatonin at a dose of 100–130 *μ*mol/L is effective as an organ storage solution component and appears to be safe. It improves the vital functions of steatotic livers, which is especially important in the shortage of healthy organs. An important limitation when using melatonin may be its short half-life and interactions resulting from combining melatonin with immunosuppressive drugs (according to the Natural Health Products Directorate). It is suggested that the melatonin bioactivity index over time is higher if it is in the form of nanoparticles (Mel-NPs) [53]. Further research is necessary to optimize solutions with melatonin.

### 4.2. Prolactin

Prolactin (mammotropin and lactotropin) is a 23 kDa polypeptide hormone, made up of 198 amino acid residues and occurring in three basic isoforms that exhibit immunoreactivity. It is a multifactorial hormone with one of the widest ranges of physiological activities. It stimulates over 300 biological processes occurring in mammalian organisms. It shows endocrine, paracrine, and autocrine activity [54, 55]. It acts as a cytokine and growth factor, neurotransmitter, immune regulator, regulator of the reproductive cycle, metabolism, and internal homeostasis. It is secreted by the eosinophil cells of the anterior pituitary, ovarian follicular cells, endometrial stromal cells, vascular endothelium cells, temporal membrane, dermal fibroblasts, prostate gland, cerebral cortex, spinal cord, lacrimal gland, sweat gland, thymus, spleen, peripheral blood lymphocytes, and adipocytes [56, 57]. Prolactin receptors are located in most of the body tissues (e.g., in the brain, cerebellum, epithelium of the nipple, liver, kidneys, and lymphocytes), determining the pleiotropic effect of the hormone [58]. Prolactin stimulates proliferation and differentiation of various types of cells (of the immune system, skin, liver, lungs, pancreas, intestines, and prostate) and inhibits apoptosis [59]. It connects with its receptor (PRLr) in the pathway of Jak2, Fyn, and Tec tyrosine kinases, SHP-2 phosphatase, Vav guanine nucleotide exchange factor, and suppressor of cytokine signalling (SOCS) [60]. Prolactin alters the properties of actin cytoskeleton and adhesion of endothelial cell monolayers in a model of mechanical damage to cell culture [61]. It has been found that it is a potent liver mitogen and proangiogenic hormone. It stimulates normal liver growth and regulates its regeneration by lowering IL-6 concentration, regulating SOCS-3 expression (suppressor of cytokine signalling), and increasing hepatocyte proliferation and angiogenesis [62].

Ryszka et al. rinsed isolated rabbit livers with Ringer's solution, which was supplemented with prolactin. The effectiveness of the modified solution was assessed on the basis of biochemical tests. They found that PRL decreased the amount of released alanine aminotransferase (ALT) and aspartate aminotransferase (AST) and slowed down their release rate, which suggests its hepatoprotective properties [4]. The results obtained by Ryszka's team were confirmed by Dolińska et al., who used an isolated porcine liver model to study the effect of prolactin in the HTK solution [5]. The combination of prolactin with cysteine (an antioxidant) in HTK reduced the amount of released transaminases (AST and ALT), lactate dehydrogenase (LDH), and lactic acid. Modification of the solution also resulted in the smaller release of K^+^ and Mg^2+^ ions and larger release of Na^+^ and Ca^2+^ ions [6]. Szulc-Musioł et al. analysed the pharmacological effectiveness of prolactin in the model of rabbit liver ischemia based on Pringle's manoeuvre. Prolactin at a dose of 100 IU/L was added to HTK (control test: HTK without prolactin), and the degree of hepatocyte damage was analysed based on the measurement of biochemical parameters (ALT, AST, LDH, GGTP, and lactates) and the histopathological examination of tissues. It was found that, in the livers rinsed with HTK with the addition of PRL, the outflow of enzymes was significantly lower compared to the control group, which suggests that prolactin inhibits the process of liver cell cytolysis [7]. Budziński et al. evaluated the effect of prolactin added to HTK on the degree of hepatocyte apoptosis in an isolated porcine liver model. Apoptosis in the collected tissue samples was assessed by TdT-mediated dUTP nick-labelling. They adopted a prevalence scale ranging from 0 to 3+, depending on the number of observed nuclei and apoptotic bodies (AB). They found that the addition of prolactin improved the protective properties of HTK, maintaining a high level of apoptosis. Prolactin may influence the stabilization of cell membranes by reducing oncotic necrosis [8]. In another study, using the same research model, Budziński et al. analysed the influence of PRL on the level of catecholamines after 12 hours of liver storage in HTK + PRL and control solutions. The amount of released dopamine and adrenaline significantly decreased after rinsing grafts with HTK modified with the addition of prolactin, which correlates with the degree of ischemic liver injury [9]. The results obtained in the above publications indicate hepatoprotective properties of prolactin.

### 4.3. Dopamine

Dopamine (molecular mass of 153.181 Da) inhibits the synthesis and secretion of prolactin by acting on specific type 2 (D_2_) receptors located in the pituitary gland. It is secreted by the hypothalamic neurons, in the region of the tuber cinereum and pituitary stalk (tuberoinfundibular dopaminergic cells—TIDA). It reaches the pituitary eosinophil cells via the pituitary portal circulation. Dopamine (3,4-dihydroxyethylamine) is a neurotransmitter belonging to catecholamines [63]. It shows autocrine and paracrine effects in peripheral tissues [64]. Dopamine receptors are located in the presynaptic and postsynaptic parts of nerve cell membranes, in the kidneys, pancreas, pulmonary alveoli, and the blood vessels of the lungs, kidneys, and heart [65–69]. There are five types of dopamine receptors D1 (D_1_ and D_5_) and D2 (D_2_, D_3_, and D_4_) [70]. Yard et al. have found that catecholamines protect cells against preservation injury by scavenging reactive oxygen species or by inhibition of reactive oxygen species production [71].

Koetting et al. investigated the efficacy of dopamine in HTK in an *ex vivo* isolated male Wistar rat liver model. Dopamine was tested at three concentrations of 10, 50, and 100 *μ*mol/L. The activities of released ALT, LDH, and GLDH were adopted as liver damage parameters. Liver samples were subjected to histopathological examination. Dopamine induced a dose-related reduction in parenchymal (ALT and LDH) and mitochondrial (GLDH) enzyme release. Bile production and tissue ATP were doubled. The histopathological examination revealed that tissue injury was significantly reduced. The highest effectiveness was obtained for the dopamine dose of 50 *μ*mol/L. The least effective dose was 100 *μ*mol/L [10]. Minor et al., based on the model proposed by Koetting [10], used hypothermic machine preservation (HMP) for liver preservation. HTK was equilibrated with 100% oxygen, and dopamine was added at 10, 50, or 100 *μ*mol/L. The livers were flushed via the portal vein and subjected to 20-hour HMP at 5 ml/min, at 4°C. No influence of dopamine on vascular resistance, oxygen uptake, or lactate production was found at any concentration. The dopamine dose of 50 *μ*mol/L proved to be optimal. There was a reduced amount of released ALT, increased bile flow, and limited lipid peroxidation (LPO). Dopamine improves functional recovery of livers [11].

### 4.4. Erythropoietin

Erythropoietin (EPO) is an endogenous hormone with a molecular mass of 34 kDa, made up of 165 amino acids. In foetal life, EPO is mainly produced in the liver (90%). In postnatal life, it is produced in 80–90% by periurethral cells and renal cortical fibroblasts and in 10–20% by hepatocytes and Kupffer cells. Small amounts of EPO are produced in the lungs, brain, testes, placenta, and retina. The physiological concentration of endogenous erythropoietin is 6–32 *μ*g/ml and has a circadian rhythm (the lowest values in the morning hours, the highest at night). Its production depends on the oxygen concentration, which is why its intensive biosynthesis is observed in hypoxemia and anaemia. It is a cytokine with autocrine and paracrine effects. In addition to the stimulation of erythropoiesis in the bone marrow, it also has antioxidant, cytoprotective, pleiotropic, anti-inflammatory, and angiogenic effects. It can regulate glucose metabolism [72–75]. It is believed that it can affect the immune system by lymphocyte activity modulation and pro- and anti-inflammatory cytokine concentration. EPO affects cells via the cytokine receptor, which consists of an extracellular domain, transmembrane domain, and intracellular domain [72]. EPO receptors are located in the kidneys, lungs, pancreas, muscle tissue, neurons, endothelial cells of the blood vessels, fibroblasts, cardiomyocytes, and many carcinomas [76]. Clinical trials indicate that EPO may affect liver regeneration [74], regulate AST activity in the ischemia-reperfusion model [73, 77–80], minimize oxidative stress and caspase-3 activity [81], and reduce nuclear factor-κB expression [82]. Erythropoietin at a dose of 500–1000 IU/kg body weight [12] has a hepatoprotective effect. Bramey et al., in turn, carried out research on the efficacy of EPO in the model of cultured rat hepatocytes and found that the hormone had no protective effect on cell damage due to hypoxia, reoxygenation, and induced apoptosis [83].

Eipel et al. investigated the effect of the addition of erythropoietin (EPO) to HTK on the functions of steatotic and lean livers in a mouse model. They found that the applied modification minimized the amount of released AST (aspartate aminotransferase), LDH (lactate dehydrogenase), and GLDH (glutamate dehydrogenase) enzymes in steatotic livers up to 50%. In addition, the modified solution ameliorated I/R-associated endothelial denudation in steatotic livers. These dependencies were not observed in lean livers. EPO reduced Erk phosphorylation in lean livers. The authors suggest that EPO can increase the effectiveness of marginal liver transplantation. The response of only steatotic livers to EPO may be the result of increased hepatocyte sensitivity to hypoxia and reoxygenation compared to lean livers. EPO in lean livers tends to accentuate tissue damage [12]. The above study does not unambiguously confirm the efficacy of erythropoietin as a component of the HTK solution.

### 4.5. Insulin

Insulin is a peptide hormone that participates in the metabolism of carbohydrates, lipids, and proteins and mediates the inhibition of their breakdown and release into the bloodstream. It is made up of 51 amino acid residues with a molecular mass of 5.76 kDa. Insulin secretion modulates the sympathetic nervous system and catecholamines. This hormone is produced by the beta cells in pancreatic islets of Langerhans containing *α*- and *β*-adrenergic receptors, the activation of which yields opposite effects. Stimulation of *α*-adrenergic receptors inhibits insulin secretion, whereas stimulation of *β*-adrenergic receptors stimulates the hormone secretion. Insulin has peripheral effects in skeletal muscle, adipose tissue, and liver cells. It stimulates the uptake of glucose by myocytes, adipocytes, and hepatocytes, inhibits gluconeogenesis, and stimulates glycogenogenesis. It is a strong anabolic hormone, conducive to the storage of metabolic reserves [84–86]. It is suggested that insulin as a component of the UW solution may stimulate the process of glycolysis and liver regeneration [30, 87]. Its administration in donors and recipients improved the graft functions by stimulating energy repletion [88]. The introduction of the hormone into the UW preservation solution is intended to stimulate the production of ATP by glycolysis during liver storage [89].

Li et al. investigated the efficacy of insulin addition to UW in a rat liver transplantation model. Isolated grafts were stored for 24 hours in hypothermia, and then their functions were tested. It was found that the addition of insulin aggravated ischemia-reperfusion injury of the liver, which may affect the inferior graft survival. An increase in the activity of ALT and AST transaminases as well as lower expression of 215 genes was observed [13]. Moreover, the addition of insulin to UW significantly reduced the ATP level, adenine nucleotide pool (TAN), and energy resources of hepatocytes. The glycogenogenesis process and lipoprotein metabolism were accelerated by increased activity of apolipoprotein C-III (Apo C-III) [14]. It can be assumed that the lack of glucose in the UW composition affected the lack of improvement in the energy potential of the liver rinsed with the insulin-enriched solution.

### 4.6. Glucagon

Glucagon is a polypeptide hormone composed of 29 amino acid residues with a molecular mass of 3.5 kDa. The normal plasma glucagon concentration is 50–150 ng/l [90]. It is secreted by alpha cells of pancreatic islets in response to the low glucose concentration and insulin, somatostatin, and fatty acid levels in the blood. The main task of glucagon is to antagonize the action of insulin. Glucagon is a stimulator of hepatic glycogenolysis, gluconeogenesis, and ketogenesis. It also has inotropic and chronotropic effects independent of the activation of *β*-adrenergic receptors [91, 92]. Glucagon action is transduced by the class B G-protein-coupled glucagon receptor (GCGR). Glucagon receptors are found in the kidney, brain, lymphoid cells of the spleen and thymus, parenchymal cells of the liver, and endothelial and Kupffer cells in the liver, heart, adipose tissue, intestinal smooth muscle tissue, and endocrine pancreatic cells [93, 94]. The attachment of glucagon to a specific receptor on the surface of hepatocytes leads to the activation of adenylyl cyclase, production of cyclic AMP (cAMP), and stimulation of protein kinase A (PKA). PKA, in turn, phosphorylates proteins that contribute to increased hepatic glucose production [95]. Normal cAMP concentration is also an important determinant of the physiological behaviour of the cytoskeleton structure and the maintenance of the physical barrier of endothelial cells [96]. The addition of glucagon to rat liver perfusates may increase cAMP concentration [97].

Minor et al. analysed the effect of glucagon added to UW on hepatocytes in an isolated rat liver model. They have found that the hormone influences the maintenance of the correct structural integrity of hepatocytes (increase in cAMP concentration and decrease in ALT activity), increases bile production in the liver, and regenerates ATP levels in tissues [15].

### 4.7. Trophic Factors

Hepatic tissue regeneration is possible through hepatocyte proliferative activity. Endogenous hepatic progenitor oval cells with exogenous multipotent bone marrow cells are also involved in this process [98]. Protein growth factors act as regulators of the proliferation process of these cells. They mediate endocrine and paracrine regulation of cell growth and differentiation. Organ perfusion and preservation solutions were modified with the addition of IGF-1 (insulin-like growth factor-1), EGF (epidermal growth factor), NGF (nerve growth factor), and HGF (hepatocyte growth factor).

Insulin-like growth factor-1 (IGF-1) belongs to polypeptide hormones with a molecular mass of 7.65 kDa. In terms of structure, it is similar to insulin and relaxin. The IGF-1 precursor is found in two isoforms, namely, IGF-1A and IGF-1B. This hormone is characterized by a pleiotropic effect [99, 100]. Its largest amount was found in liver cells (50–100 times more than in other tissues). IGF-1 produced in the liver has an endocrine effect, whereas the one synthesized in other organs (kidneys, lungs, heart, testes, brain, thyroid, gonads, and large intestine) has an autocrine and/or paracrine effect [101, 102]. It participates in the regulation of cell differentiation and proliferation. It shows biological activity similar to insulin. It stimulates glucose oxidation and lipogenesis and inhibits lipolysis [103]. It promotes the growth of many body tissues, including postnatal growth, and regulates homeostasis. It stimulates regulatory T lymphocytes (Treg) to produce anti-inflammatory cytokines (IL-10). IGF-1 interacts with the cell by binding to IGF-1R membrane receptors (insulin-like growth factor-1 receptor). This receptor mediates the mitogenic and antiapoptotic activities of IGF-1 and affects cell transformation. This receptor was found inter alia in liver cells (Browicz–Kupffer cells, myofibroblasts, and stellate cells) and cells of the lymphoid organs [104–107]. IGF-1 concentration in blood serum depends on age and gender. Its highest increase is observed during puberty, and it significantly decreases after the age of 25. In girls aged about 14 years, IGF-1 concentration is ∼410 *μ*g/l, whereas in boys aged about 15 years IGF-1 concentration is ∼382 *μ*g/l. Changes in IGF-1 concentration depend on the growth hormone concentration [108].

The IGF-1 receptor is essential for the biological activity of the epidermal growth factor (EGF) [109]. EGF stimulates angiogenesis and mitogenesis and regulates the secretion of collagenesis. The EGFR receptor affects biological processes occurring in cells: their growth, differentiation, adhesion, apoptosis, and DNA repair [110]. NGF (nerve growth factor) is in turn a polypeptide with a molecular mass of 26 kDa and belongs to trophic factors (TFs) affecting the population of neurons of the central and peripheral nervous system. The influence of NGF on the immune system (stimulation of the growth and differentiation of B and T lymphocytes) and endocrine system (stimulation of the differentiation of pancreatic *β*-cells) has been confirmed. NGF affects cells by binding to membrane receptors: NTRK1 and TNFRSF1B [111, 112]. HGF (hepatocyte growth factor) belongs to the plasminogen proteins and consists of two subunits: the *α*-subunit with a molecular mass of 60 kDa and the *β*-subunit with a molecular mass of 30 kDa [113]. It has an autocrine and/or paracrine effect. It is produced by fibroblasts, epithelial and endothelial cells, fat-accumulating cells in the liver, and marrow stromal cells [114]. HGF after binding to the MET receptor is involved in mitogenesis, morphogenesis, cell growth and proliferation, tissue regeneration, inhibition of apoptosis, and repression of intercellular adhesion [115].

Zaouali's team studied the effect of the addition of insulin-like growth factor-1 (IGF-1, 10 *μ*g/l) on the efficacy of the IGL-1 solution in an isolated rat liver model. They found an increase in the activity of AKT (serine-threonine kinase) and eNOS (endothelial nitric oxide synthase) and inhibition of TNF-*α* proinflammatory cytokine release [16]. In turn, the addition of the epidermal growth factor (EGF-10 *μ*g/l) to the IGL-1 solution in an isolated rat liver model slowed down the release of aminotransferases, increased bile production, and, as a consequence, prevented energy metabolism deterioration, mitochondrial damage, oxidative stress, and cytokine IL-1 beta release [17]. The authors combined the action of IGF-1 and EGF in the UW solution in steatotic and nonsteatotic rat liver models. They found that the addition of EGF and IGF-1 (separately or in combination) to UW reduced hepatic injury and improved function in both liver types. EGF and IGF-I upregulated AKT. AKT protected both liver types, inactivating GSK3*β* in nonsteatotic livers and inducing PPARγ overexpression in steatotic livers [18]. Ambiru et al. added the trophic factors (TF) to the University of Wisconsin solution. They found that the combination of growth factors (IGF-1, EGF, and NGF) resulted in a significantly better 5-day survival (57%) compared to using UW alone (14%). Moreover, ATP content was significantly higher than in grafts preserved in UW. The obtained results indicate that EGF can induce hepatocyte proliferation and increase DNA synthesis in hepatocytes. IGF-1 provides inhibition of apoptosis induced by ischemia-reperfusion injury. NGF is related to tissue remodelling in the liver. The absence of trophic factors may cause apoptosis, cell cycle arrest, and cell death [19]. Further studies are necessary to achieve a comprehensive understanding of the system of trophic factors and their receptors. Takeda et al. analysed the effect of human recombinant hepatocyte growth factor (hrHGF) added to UW on the graft function in a rat fatty liver model. They assessed ultrastructural alteration of hepatocytes, sinusoidal architecture, endothelial cells (SECs), hyaluronic uptake rate (HUR), and alanine aminotransferase (ALT) level. The hepatocyte injury and SECs developed more rapidly than in the control solution, i.e., UW. The results obtained suggest hepatoprotective effects of hrHGF [20, 21].

### 4.8. Relaxin

Relaxin is a polypeptide hormone with a molecular mass of 6 kDa and a structure similar to insulin and insulin-like growth factor. However, it does not activate insulin receptors and has a different multidirectional effect on tissues. It interacts through the endocrine, paracrine, and autocrine pathways. Relaxin-2 is present in the ovaries, uterus, testes, prostate gland, brain, cardiovascular system, skin, lungs, liver, and kidneys [116, 117]. It activates the LGR7 and LGR8 receptors belonging to the transmembrane receptors, coupled to the G protein. It participates in cell proliferation and differentiation. It relaxes the walls of blood vessels as a result of stimulation of nitric oxide production in endothelial cells. It has cytoprotective and anti-inflammatory effects. It inhibits fibrosis processes in damaged organs (including the liver) by activating metalloproteinases [118–121].

Boehnert et al. analysed the efficacy of relaxin in an isolated perfused rat liver model. Relaxin was added to the UW [22] and HTK [23] solutions and its effect during graft preservation and reperfusion was studied. They found a protective effect of relaxin. The activity of malonyldialdehyde (MDA, end product of lipid peroxidation) and myeloperoxidase (MPO, marker for accumulation of neutrophil granulocytes) was lower compared to the control group. Relaxin affects decreased peroxidation and increased oxygen.

### 4.9. Prostaglandin E1

Prostaglandin E-1 (PGE-1) has a molecular mass of 354.48 Da. It belongs to paracrine hormones that have high biological activity. It is formed from arachidonic acid in the cyclooxygenase process. It is found in all tissues, organs, and body fluids, except for red blood cells. It exhibits a short half-life, which is why it usually acts locally. It takes part in the regulation of homeostasis. It has a relaxant effect on smooth muscles, especially the ductus arteriosus muscles [122]. It stimulates the activity of adenylyl cyclase in platelets. It demonstrates the ability to bind to cell surface receptors increasing the concentration of cAMP (cyclic adenosine monophosphate), which is involved in the regulation of biochemical processes as an element of signal transduction. In addition, it inhibits the release of Ca^2+^ ions [123]. Stellate cells of the liver under the influence of prostaglandins have the ability to relax causing diastole [124]. PGE-1 secretion in Kupffer cells in response to ischemia/reperfusion has been detected [125]. As a result of the release of growth factors, TNF-*α* and prostaglandins, appropriate receptors are activated on the surface of hepatocytes, and proliferation of these cells is increased through the NF-κB pathway [126]. It has been found that PGE-1 has a cytoprotective, regenerative effect, increases the liver perfusion, inhibits platelet aggregation and protease, and minimizes the production of free oxygen radicals [127, 128].

Aliosmanoglu et al. studied the effect of prostaglandin E-1 (PGE-1) on the damage of rat livers perfused with the University of Wisconsin (UW) solution or the histidine-tryptophan-ketoglutaric solution (HTK). The authors assessed the biochemical and histopathological parameters of the perfusates and tissues collected. They stated that PGE-1 improved the activity of grafts. Cell damage was smaller compared to the other test groups [24]. Morioka et al. [25] added PGE-1 at a dose of 1 *μ*g/mL to the composition of HTK and Ringer's lactate. The solution effectiveness was analysed based on a rat fatty liver model. The hormone improved the rate of graft survival (75% 7-day survival) and decreased ALT activity and hyaluronic acid concentration. The results obtained in the above studies indicate the protective effect of PGE-1 during ischemia/reperfusion of the liver. The hormone improves the vital functions of rat fatty livers.

## 5. Barriers to Clinical Trials

The studies on the effectiveness of commercial hormone-modified preservation solutions presented in the review have been performed in animal models, representatives of the mammal group. In the available literature, there was no information on completed or ongoing clinical trials on this subject until May 31, 2019. We suggest that there are several reasons that may constitute a barrier to these tests. Liver donors are people of a wide age range (from children to the elderly), in whom the occurrence of comorbidities increases with age. In the animal models, healthy/young animals or mammals with induced liver disease (steatotic liver model) were selected for testing, which is difficult to refer to acquired human livers. Species differences in liver anatomical structure should also be taken into account. The most similar in terms of size and physiologically similar to human livers are organs derived from domestic pigs, which were used in the model of prolactin [5, 6, 8, 9] and IGF-1/EGF/NGF efficacy [19]. The effects of hormone administration *in vivo* depend not only on the mammalian species, but also on its sex, age, biopharmaceutical dose and dosage form, time of day and season of its administration, and time of use [129]. The proposed hormone doses were not optimized in most studies. Only in some studies, in which the effect of biopharmaceutical concentration on the effectiveness of the modified solution was analysed, it was found that the increase in hormone concentration did not correlate with its higher efficiency [10, 11]. The possible interactions between components of preservation solutions and hormones should also be considered. Each, even the smallest change in the chemical structure and physicochemical properties of the substances included in the solution affects its effectiveness. Hormones may also interact with pharmaceutical preparations used in recipients during and after liver transplantation (e.g., immunosuppressants). In addition, they may affect the transplanted liver function in the long term (the performed studies were short term) [130].

## 6. Conclusion

Based on the performed analyses on the effectiveness of hormone-modified organ perfusion and preservation solutions, it can be concluded that it is possible to develop new fluid formulations that enable to preserve or improve vital functions of the liver, including marginal grafts. Further research in this direction is still necessary. It will allow to get to know the mechanism of action of these substances on the liver and develop new, prospective solutions protecting the organ during ischemia/reperfusion. It will also allow for the development of effective systemic therapy with limited side effects.

A great challenge and future for commercial preservation solutions containing biopharmaceuticals is the development of a formulation with optimal pharmacokinetics and hormone biodistribution, focused on high effectiveness in minimizing ischemic-reperfusion injury. Ensuring adequate physicochemical stability of the solution during its storage and use is also an extremely important element. The possibility of using hormone analogues with a prolonged half-life seems to be promising. A particularly interesting strategy is the use of hormones in the form of nanocarriers, which could affect the kinetics of their release (obtaining fast and/or prolonged or controlled action), increase physicochemical stability, ensure delivery to a specific site of action, and minimize the risk of interactions. Potential hormone delivery systems may be nanoemulsions, liposomes, and polymer micelles. They allow to increase biopharmaceutical bioavailability and show the ability for selective accumulation and retention in tissue for a long time.

Due to security issues, which are the priority when implementing new techniques into therapeutic regimens, it is important to analyse the long-term effect of the hormone dosage form on the recipient's liver/body.

## Figures and Tables

**Table 1:** Strategies based on modification to preservation solutions.

Author, year of publication	Hormone	Species	Preservation solution modification/cold ischemia/key settings of the experiment	Outcome measures, *n* (intervention, I/control, C)	Hormone dose	Drugs/substances used simultaneously/dose	Effects of hormone
Zaouali et al., 2011 [1]	Melatonin	Rat	IGL-1/24 h, 4°C/normothermic reperfusion: 2 h, 37°C	I: IGL + MEL; *n* = 16 C1: Ringer's lactate; *n* = 16 C2: IGL-1; *n* = 16 C3: UW; *n* = 16	100 *μ*mol/L	—	(i) Lower transaminase levels (AST/ALT) (ii) Higher bile production (iii) Higher BSP clearance (iv) Reduced vascular resistance (v) Increased nitrites/nitrates (vi) Prevention of oxidative stress
Zaouali et al., 2013 [2]		Rat	IGL-1/24 h, 4°C/normothermic reperfusion: 2 h, 37°C	I: IGL + MEL + TMZ; C1: Ringer's lactate; C2: IGL-1; C3: UW; C4: IGL + MEL + TMZ + araA	100 *μ*mol/L	TMZ 10^−3^ *μ*m/L	(i) Decreased GRP78, pPERK, and CHOP activation after reperfusion (ii) Inhibition of AMPK induced an increase in ER stress (iii) Significant reduction in autophagy
Gunal et al., 2010 [3]		Rat	UW/48 h, 4°C	I: UW + MEL; *n* = 10 C: UW; *n* = 10	30 mg/L	—	(i) Lower transaminase levels (LDH, AST, and ACP) (ii) Induced heat-shock protein (HSP 70) (iii) Decreased lipid peroxidation (iv) Prevented Kupffer cell activation and inflammation

Ryszka et al., 2004 [4]	Prolactin	Rabbit	Ringer/24 h, 4–6°C	I: Ringer + PRL; *n* = 5 C: Ringer; *n* = 5	100 IU/L	—	(i) Lower transaminase levels (AST and ALT) (ii) Slowing the rate of transaminase release
Dolińska et al., 2011 [5]		Pig	HTK/24 h, 4–6°C	I: HTK + PRL; *n* = 5 C: HTK; *n* = 5	100 IU/L	—	(i) Lower transaminase levels (AST and ALT) (ii) Slowing the rate of transaminase release
Ryszka et al., 2011 [6]		Pig	HTK/24 h, 4–6°C/cold reperfusion	I: HTK + PRL; *n* = 6 C: HTK; *n* = 6	3 IU/L	Cys 0.3 mmol/L	(i) Lower levels of AST, ALT, LDH, and lactic acid (ii) Less release of K^+^, Mg^2+^ (iii) Greater release of Na^+^, Ca^2+^
Szulc-Musioł et al., 2018 [7]		Rabbit	HTK/in situ	I: HTK + PRL; *n* = 10 C: HTK; *n* = 10	2.5 *μ*g/g liver	—	(i) Inhibition of liver cell cytolysis
Budziński et al., 2011 [8]		Pig	HTK/12 h, 4°C	I: HTK + PRL; *n* = 6 C: Ringer; *n* = 6 C: UW; *n* = 6 C: HTK; *n* = 6	20 IU/L	—	(i) High apoptosis level
Budziński et al., 2011 [9]		Pig	HTK /12 h, 4°C	I: HTK + PRL; *n* = 6 C: Ringer; *n* = 6 C: UW; *n* = 6 C: HTK; *n* = 6	20 IU/L	—	(i) Significantly decreased dopamine and adrenaline concentrations

Koetting et al., 2010 [10]	Dopamine	Rat	HTK /18 h, 4°C/normothermic reperfusion: 2 h, 37°C	I: HTK + DA; *n* = 6 C: HTK; *n* = 6	10 *μ*mol/L 50 *μ*mol/L 100 *μ*mol/L	—	(i) Reduction in ALT, LDH, GLDH release (ii) Reduced histologic signs of tissue injury (iii) Doubled bile production and tissue ATP
Minor et al., 2011 [11]		Rat	HTK /20 h, 4°C/HMP, normothermic reperfusion: 2 h, 37°C	I: HTK + DA; *n* = 6 C: HTK; *n* = 6	10 *μ*mol/L 50 *μ*mol/L 100 *μ*mol/L	—	(i) Reduction in ALT release (ii) Enhanced bile flow (iii) Reduced lipid peroxidation

Eipel et al., 2012 [12]	Erythropoietin	Mouse	HTK /24 h, 4°C/normothermic reperfusion: 2 h	I: HTK + EPO; *n* = 6 C: HTK; *n* = 6 C: livers without cold storage; *n* = 6	10 IU/mL	—	(i) Prevented induced denudation of the endothelial lining in steatotic livers, but aggravated in lean livers (ii) Enzyme (AST, LDH, GLDH) release reduced to 50% in steatotic livers, but not in lean livers (iii) Steatotic livers presented with lower oxygen consumption than lean livers (iv) Reduced MAPK-dependent Erk phosphorylation in lean livers

Li et al., 2003 [13]	Insulin	Rat	UW/24 h, 4°C	I: UW + Ins; *n* = 14 C: UW; *n* = 20	40 IU/L	—	(i) Higher transaminase levels (AST and ALT) (ii) Repressed expression of 215 genes (iii) Exacerbated graft ischemic injury
Li et al., 2004 [14]		Rat	UW/24 h, 0–4°C	I: UW + Ins; *n* = 5 C: UW; *n* = 5	40 IU/L	—	(i) Deteriorated energy regeneration (ii) Acceleration of lipoprotein metabolism through upregulation of the activity of apolipoprotein C‐III (Apo C‐III) (iii) Inhibition of the insulin‐like growth factor‐binding protein‐1 pathway

Minor et al., 1998 [15]	Glucagon	Rat	UW/24 h, 4°C/normothermic perfusion: 45 min, 37°C	I: UW + Gluc; *n* = 5 C: UW; *n* = 5	0.5 *μ*g/mL	—	(i) Enhanced endogenous cAMP signal (ii) Reduction in ALT release (iii) Threefold increase in hepatic bile production (iv) Restored ATP tissue levels

Zaouali et al., 2010 [16]	IGF-1	Rat	IGL-1/24 h, 4°C/warm reperfusion: 2 h, 37°C	I: IGL-1 + IGF-1; *n* = 8 C: IGL-1, *n* = 8	10 *μ*g/L	—	(i) Lower transaminase levels (ALT, AST) (ii) Increased bile clearance (iii) Reduction in vascular resistance (iv) Activation of AKT (iv) Constitutive endothelial nitric oxide synthase

Zaouali et al., 2010 [17]	EGF-1	Rat	IGL-1/24 h, 4°C/warm reperfusion: 2 h, 37°C	I: IGL-1 + EGF-1; *n* = 8 C: IGL-1, *n* = 8	10 *μ*g/L	—	(i) Lower transaminase levels (ii) Greater bile production (iii) Ameliorated flow rates

Zaouali et al., 2010 [18]	IGF-1 EGF	Rat	UW/24 h, 4°C/normothermic reperfusion: 2 h, 37°C	I1: UW + IGF-1; *n* = 16 I2: UW + EGF; *n* = 16 I3: UW + IGF-1 + EGF; *n* = 16 C: UW; *n* = 16	10 *μ*g/L_IGF-1_ 10 *μ*g/L_EGF_	AKT inhibitor 1.5 mg/L	(i) IGF‐I could be a more appropriate clinical therapy than EGF (ii) EGF and IGF‐1 upregulated AKT (iii) EGF and IGF-I (separately or in combination) reduced hepatic injury and improved survival in recipients
Ambiru et al., 2004 [19]	IGF-1 EGF NGF	Pig	UW/18 h, 4°C/cold reperfusion	I: UW + IGF-1 + EGF + NGF; *n* = 7 C: UW; *n* = 7	10 *μ*g/L_EGF_ 10 *μ*g/L_IGF-1_ 20 *μ*g/L_(NGF)-*β*_	Bactenecin: 1 mg/L SP: 2.5 mg/L	(i) Transaminases (AST, ALT) were comparable between the two groups (ii) Higher ATP levels (iii) Less haemorrhagic necrosis (iv) Cold ischemic time extended to 18 h

Takeda et al., 1999 [20]	hrHGF	Rat	UW	I: UW + hrHGF C: UW	0.3 *μ*g/mL	100 *μ*g/body hrHGF was injected	(i) Reduced injury in SEC (ii) Prevented expansion of fatty droplets (iii) Protective effect
Takeda et al., 2003 [21]		Rat	UW/24 h, 4°C	I: UW + hrHGF C: UW	0.1 *μ*g/mL or 1 *μ*g/mL	—	(i) Decreased transaminase levels (ii) Diminished hepatocellular damage in histological examination

Boehnert et al., 2005 [22]	hr relaxin-2	Rat	UW/3.5 h, 4°C or 3.5 h, 20°C/cold reperfusion: 4°C or warm reperfusion: 20°C	I: UW + hrRLX-2; *n* = 10 C: UW; *n* = 10	32 ng/mL (reperfusion solutions) 64 ng/mL (preservation solution)	—	(i) Decreased MDA activity (ii) Decreased MPO activity
Boehnert et al., 2008 [23]		Rat	HTK/5 h, 4°C or 5 h, 20°C/cold reperfusion: 4°C or warm reperfusion: 20°C	I: HTK + hrRLX-2; *n* = 20 C: HTK; *n* = 10	64 ng/mL	—	(i) Decreased MDA activity (ii) Decreased MPO activity

Aliosmanoglu et al., 2013 [24]	Prostaglandin E-1	Rat	HTK/12 h, 4°C UW/12 h, 4°C	I1: HTK + PGE-1; *n* = 6 I2: UW + PGE-1; *n* = 6 C1: Ringer's lactate; *n* = 6 C2: HTK; *n* = 6 C3:UW; *n* = 6	20 *μ*g/kg	—	(i) Decreased transaminase levels (ii) Decreased pathologic injury
Morioka et al., 2003 [25]		Rat	HTK/2 h or 6 h, 4°C Ringer's lactate/2 h or 6 h, 4°C	I1: HTK + PGE-1; Ringer's lactate + PGE-1 C1: fatty livers + 0.3 mg/kg FK506 C2: fatty livers	1 *μ*g/mL	—	(i) 75% 7-day survival (ii) Decreased ALT and hyaluronic acid levels (iii) Reduced tissue injury

TMZ, trimetazidine; MEL, melatonin; araA, adenine 9‐*β*‐D‐arabinofuranoside, an inhibitor of AMPK; PRL, prolactin; Cys, cysteine; ALT, alanine aminotransferase; AST, aspartate aminotransferase; LDH, lactate dehydrogenase; GLDH, glutamate dehydrogenase; BSP, sulfobromophthalein clearance; GRP78, 78-kDa glucose regulated protein; CHOP, C/EBP homologous protein; ER, endoplasmic reticulum; PERK, PKR-like ER kinase; AMPK, AMP-activated protein kinase; EPO, erythropoietin; apo C‐III, apolipoprotein C‐III; Gluc, glucagon; DA, dopamine; IGF-1, insulin-like growth factor; AKT, protein kinase; EGF, epidermal growth factor; NGF, nerve growth factor; SP, substance P; hrHGF, human recombinant hepatocyte growth factor; SEC, sinusoidal endothelial cells; MDA, malonyldialdehyde; MPO, myeloperoxidase; RLX, relaxin; HMP, hypothermic machine preservation; PGE-1, prostaglandin E-1; FK506, tacrolimus hydrate.

**Table 2:** Composition of preservation solutions.

Component	Viaspan	IGL-1	HTK
IC/EX	IC	EX	EX
*Electrolytes (mmol/l)*			
Potassium	125	25	10
Sodium	29	120	15
Calcium	—	—	0.015
Magnesium	5	5	4
Chloride	20	—	32
Colloids (g/L)			
HES	50	—	—
PEG-35	—	1	—

*ROS scavengers (mmol/l)*			
Allopurinol	1	1	—
Glutathione	3	3	—
Mannitol	—	—	30
Tryptophan	—	—	2

*Buffers (mmol/l)*			
Histidine	—	—	198
KH_2_PO_4_	25	25	—

*Impermeants (mmol/l)*			
Lactobionate	100	100	—
Raffinose	30	30	—

*Additives (mmol/l)*			
Adenosine	5	5	—
Ketoglutarate	—	—	1
pH	7.4	7.4	7.2
Viscosity (mm^2^/s)	3.16	1.25	1.00
Osmolality mOsm/kg H_2_O	320	290	310

IC, intracellular; EX, extracellular.
